# Pulmonary Oxidative Stress Is Increased in Cyclooxygenase-2 Knockdown Mice with Mild Pulmonary Hypertension Induced by Monocrotaline

**DOI:** 10.1371/journal.pone.0023439

**Published:** 2011-08-05

**Authors:** Francesca Seta, Mahboubeh Rahmani, Patricia V. Turner, Colin D. Funk

**Affiliations:** 1 Department of Biomedical and Molecular Sciences, Queen's University, Kingston, Ontario, Canada; 2 Department of Pathobiology, University of Guelph, Guelph, Ontario, Canada; Stanford University, United States of America

## Abstract

The aim of this study was to examine the role of cyclooxygenase-2 (COX-2) and downstream signaling of prostanoids in the pathogenesis of pulmonary hypertension (PH) using mice with genetically manipulated COX-2 expression. COX-2 knockdown (KD) mice, characterized by 80–90% suppression of COX-2, and wild-type (WT) control mice were treated weekly with monocrotaline (MCT) over 10 weeks. Mice were examined for cardiac hypertrophy/function and right ventricular pressure. Lung histopathological analysis was performed and various assays were carried out to examine oxidative stress, as well as gene, protein, cytokine and prostanoid expression. We found that MCT increased right ventricular systolic and pulmonary arterial pressures in comparison to saline-treated mice, with no evidence of cardiac remodeling. Gene expression of endothelin receptor A and thromboxane synthesis, regulators of vasoconstriction, were increased in MCT-treated lungs. Bronchoalveolar lavage fluid and lung sections demonstrated mild inflammation and perivascular edema but activation of inflammatory cells was not predominant under the experimental conditions. Heme oxygenase-1 (HO-1) expression and indicators of oxidative stress in lungs were significantly increased, especially in COX-2 KD MCT-treated mice. Gene expression of NOX-4, but not NOX-2, two NADPH oxidase subunits crucial for superoxide generation, was induced by ∼4-fold in both groups of mice by MCT. Vasodilatory and anti-aggregatory prostacyclin was reduced by ∼85% only in MCT-treated COX-2 KD mice. This study suggests that increased oxidative stress-derived endothelial dysfunction, vasoconstriction and mild inflammation, exacerbated by the lack of COX-2, contribute to the pathogenesis of early stages of PH when mild hemodynamic changes are evident and not yet accompanied by vascular and cardiac remodeling.

## Introduction

Prostacyclin (PGI_2_) is a potent vasodilator and platelet inhibitor produced in blood vessels by the enzymatic activity of cyclooxygenases (COX-1 and COX-2) and prostacyclin synthase (PGIS) [1]. PGI_2_ has been shown in vitro [1] and in vivo [2], [3] to modulate the vasoconstrictor and platelet aggregatory activities of thromboxane A_2_ (TXA_2_), a COX-derived prostanoid produced mainly by activated platelets via COX-1 during hemostasis. A disrupted interplay between PGI_2_ and TXA_2_ levels has been implicated in the pathogenesis of pulmonary hypertension (PH), a severe condition characterized by irreversible remodeling of pulmonary resistive vessels, increased pulmonary vascular tone and in situ thrombosis [4], [5], [6]. PGIS is down-regulated in patients with PH [7] and other chronic lung diseases [8] and transgenic animal models, over-expressing PGIS or with deletion of the PGI_2_ receptor (IP), have unequivocally demonstrated a protective role of PGI_2_ in settings of PH [9], [10], [11]. To date, PGI_2_ analogs are among the few therapeutic options available to improve hemodynamic parameters and survival of patients with PH. A direct vasodilatory effect on pulmonary vasculature, modulation of arterial thrombosis and inhibition of vascular remodeling, can all account for these beneficial effects [12]. On the other hand, COX-1 inhibitors or TXA_2_ receptor antagonists improve PH only partially since other mechanisms of platelet aggregation, via ADP, collagen, serotonin and thrombin, may sustain intra-pulmonary arterial thrombosis and progression of the disease, even in settings of profound TXA_2_ inhibition [13].

COX-2 inhibitors (coxibs) represent a subgroup of non-steroidal anti-inflammatory drugs (NSAID) that target selectively COX-2 and spare almost completely COX-1 activity. Administration of celecoxib, one of the first COX-2 inhibitors developed, to healthy humans profoundly suppressed in vivo PGI_2_ biosynthesis leaving TXA_2_ production intact [14]. Moreover, coxibs consistently increased the risk of cardiovascular events, related mostly to thromboembolic events, compared to non-selective NSAIDs or placebo [15]. In hypoxia-induced PH models, administration of COX-2 inhibitors [16] or genetic knock out of COX-2 [17], [18], [19] decreased PGI_2_ levels, failed to reduce hypoxia-induced thromboxane production and exacerbated the rise in pulmonary pressures and vascular remodeling.

In the present study, we employed a novel mouse model of COX-2 inhibition, that mimics coxib administration, characterized by a knock down of COX-2 (COX-2 KD) expression (≈80%) with disrupted PGI_2_ production, but with intact COX-1-derived TXA_2_ biosynthesis, and increased tendency to thrombogenesis [20], in monocrotaline (MCT)-induced PH.

The MCT-induced PH model is well established in rats but it remains controversial in mice since the severity of MCT-induced PH and associated pulmonary and cardiac histopathological changes are variable [21], [22], [23], [24], [25]. This is attributed mainly to species- and strain-specific differences in hepatic cytochrome P450 enzymes required for MCT biotransformation into the active MCT pyrrole, rendering this model less reproducible in mice than in rats [26], [27]. However, more recently, repeated MCT administration at high doses (600 mg/kg body weight) and/or for prolonged treatment (8 weeks) than in previously employed studies, appears to more consistently and reproducibly induce PH in mice [28], [29], [30], [31]. Despite hypoxia being most commonly used in mice as a model of PH, we opted for the use of MCT because, in contrast to hypoxia-induced PH. MCT-induced PH is characterized by increased pulmonary vascular permeability and remodeling consequent to direct injury of MCT to the alveolar capillary endothelium [32], [33]. In addition, this model has not previously been studied with COX-2 modulation. Lastly, MCT has been reported to increase pulmonary resistance in dogs, at least in part, by increasing arterial thrombosis, associated with increased circulating thromboxane levels, that is ameliorated after PGI_2_ infusion [34].

In this study, we aimed to elucidate the role of COX-2, an abundant source of PGI_2_, in the pathogenesis of PH by comparing COX-2 KD mice and WT controls after pulmonary endothelial injury induced by MCT administration. Here, we describe our findings and the limitations of using MCT as a model of PH in mice.

## Results

### Monocrotaline induced mild pulmonary hypertension (PH) in WT and COX-2 KD mice

Preliminary experiments aimed to assess the feasibility of consistently inducing PH in mice by monocrotaline (MCT) administration and the dosing regimen required. MCT in the range 50–300 mg/kg BW, weekly for 4 weeks, failed to significantly increase right ventricular pressure, used as an index of pulmonary artery pressure, in mice. At 300 mg/kg BW MCT dose, right ventricular pressures showed a modest increase versus saline treatment but did not reach statistical significance (WT/saline: 6.6±0.8, n = 4; COX-2KD/saline: 6.7±0.7, n = 6; WT/MCT: 9.8±0.9, n = 5; COX-2KD/MCT: 8.2±2.7 mmHg, n = 3). Moreover, immunolabeling of lung sections with α-smooth muscle actin did not reveal any significant increases in pulmonary arteriole muscularization in MCT-treated mice, compared to the saline-treated group (data not shown). Therefore, in the current study, we increased the MCT dose to 600 mg/kg once weekly for 10 weeks. A similar regimen has been recently used by several investigators to consistently induce PH in mice [28], [29], [30], [31], [35].

**Figure 1:**
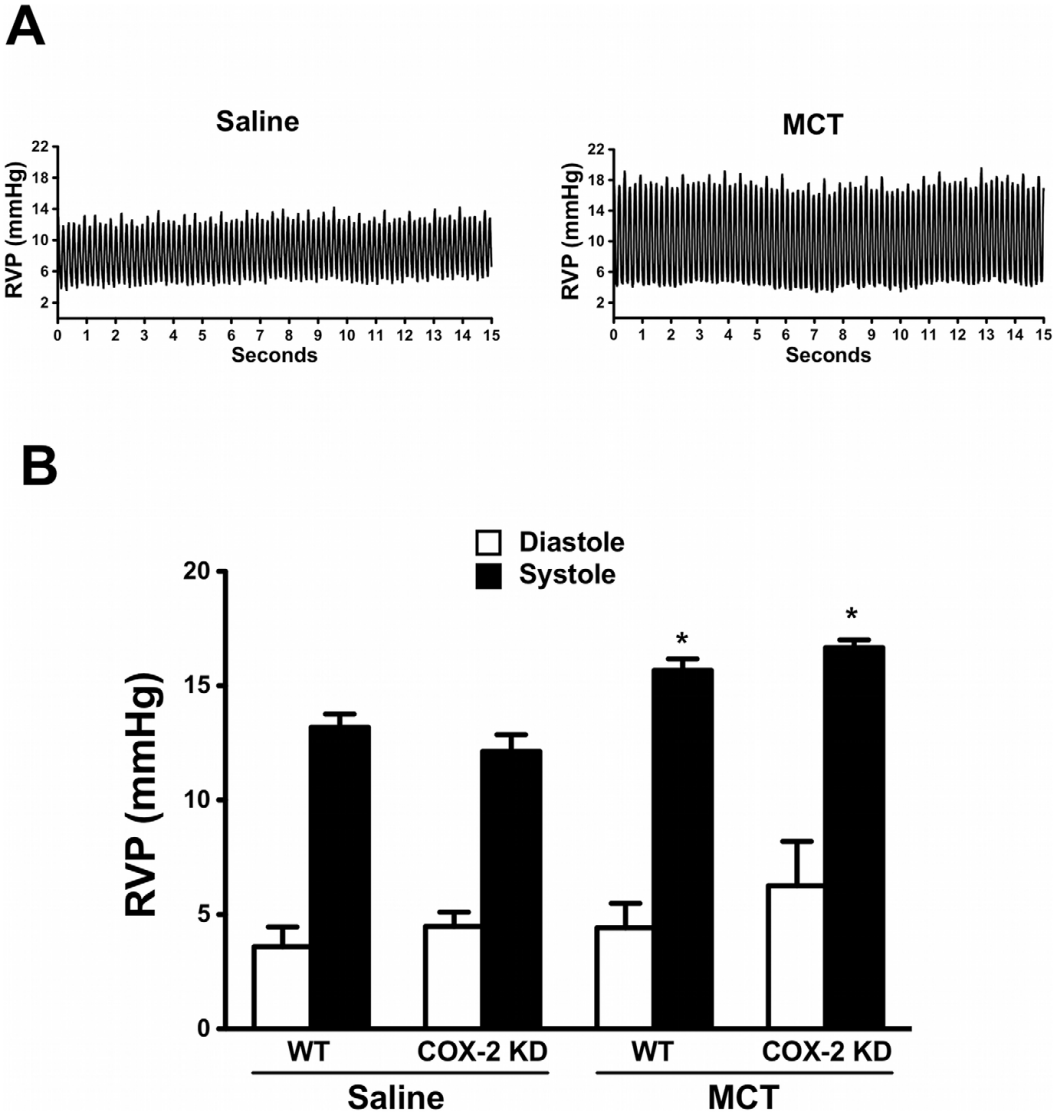
Monocrotaline-induced pulmonary arterial hypertension in mice. A. Representative right ventricular pressure (RVP) tracings recorded after 10 wk of monocrotaline (MCT) or saline administration, by inserting a pressure transducer directly into the right ventricle. B. Right ventricular pressures (RVP) were calculated from tracings as in panel A, by averaging 15 s intervals of continuous recording. WT/saline, n = 6; WT/MCT, n = 5; COX-2 KD/saline, n = 5; COX-2 KD/MCT, n = 3. mmHg, millimeters of mercury. *, p<0.05 vs saline.

MCT treatment induced a consistent body weight loss compared to saline-treated mice (change BW: WT/saline: +7.7±1.2%, n = 6; COX-2 KD/saline: +6.3±2.1%, n = 6; WT/MCT: −10.5±3.2%, n = 11; COX-2 KD/MCT: -9.7±2.8%, n = 5; p<0.05 saline vs MCT; n indicates number of animals that completed the study) over the 10-week study period. Unexpectedly, 14 of 19 COX-2 KD mice died or experienced duress requiring euthanasia compared to only 3 of 14 MCT-treated WT mice (overall survival rate: 79% for MCT-treated WT, n = 14; 26% for MCT-treated COX-2 KD, n = 19; 100% saline-treated WT, n = 6 and 100% saline-treated COX-2 KD, n = 6; p = 0.0006, WT-MCT vs COX-2 KD-MCT, Mentel-Cox test) (Fig. S1). Post-mortem histopathological analysis on 2 WT and 3 COX-2 KD MCT-treated mice that required euthanasia revealed acute hepatic necrosis that was more pronounced in COX-2 KD mice. MCT at 600 mg/kg BW induced a modest but significant increase in right ventricular end systolic pressure in comparison to saline-treated mice (WT/saline: 13.2±0.5, n = 6; COX-2KD/saline: 12.1±0.6, n = 5; WT/MCT: 16.1±0.3, n = 5; COX-2 KD/MCT: 16.7±0.3 mmHg, n = 3; mean±SEM, p<0.05 MCT vs saline; **Figure 1A and B**), with no increase in right ventricular end diastolic pressure (WT/saline: 3.6±0.8, n = 6; COX-2KD/saline: 4.5±0.6, n = 5; WT/MCT: 5.4±0.4, n = 5; COX-2KD/MCT: 6.2±1.9 mmHg, n = 3; mean±SEM, NS; **Figure 1A and B**). Doppler analysis at the level of the pulmonary valve, recorded by ultrasonography on lightly anesthetized mice (heart rate 400–500 bpm), demonstrated an increase in the pulmonary arterial blood flow gradient and velocity in systole after MCT treatment (**Table 1**). Left ventricular function and dimensions were similar in all treatment groups (**Table S1**). These results are consistent with an increased right ventricular afterload during systole indicating that MCT increased pulmonary artery pressure mainly by increasing pulmonary vascular resistance, with no impairment of left ventricular function.

**Table 1:** Pulmonary artery blood velocity is increased after MCT treatment.

	VTI,cm	Mean Gradient, mmHg	Peak Gradient, mmHg	Mean Velocity,mm/s	PeakVelocity, mm/s
**WT/saline (n = 6)**	2.52±0.2	0.28±0.04	1.4±0.19	260.8±18.8	583.8±38
**WT/MCT (n = 11)**	3.06±0.18*	0.5±0.09*	2.52±0.51*	343±28*	764±65*
**COX-2KD/saline (n = 6)**	2.53±0.29	0.28±0.03	1.42±0.17	261±16	590±35
**COX-2KD/MCT (n = 6)**	3.84±0.4* #	0.81±0.15*	4.45±0.94*	438±43*	1027±108* #

Velocity-time integral (VTI, cm), mean and peak gradient (mmHg) and mean and peak velocity (mm/s) of blood flow in the pulmonary artery were measured from Doppler waveforms acquired by ultrasound imaging. Number of mice for each group is in parentheses. Mean±SE;

*p<0.05 vs saline;

# p<0.05 vs WT/MCT.

### Vasoconstrictors in lungs after MCT

We investigated the expression of endothelin-1 receptor A (ETR-A) as one of the possible molecular mechanisms contributing to increased pulmonary vascular resistance in response to MCT. Endothelin-1 is the most potent and long-lasting endogenous vasoconstrictor produced by endothelial cells and is a mitogen for vascular smooth muscle cells [36], [37], [38]. Endothelin-1 circulating levels are increased in patients with PH [39] and ETR antagonists are commonly used to treat PH [40], [41]. We found that ETR-A gene expression was significantly induced in response to MCT, as measured by quantitative PCR on lung tissue homogenates (fold-increase vs saline: 2.3±0.4 in WT/MCT, n = 8; 2.2±0.6, n = 7 in COX-2 KD/MCT; p<0.05). Therefore, in MCT-induced PH in mice, the endothelin-1 signaling pathway appears to be upregulated and may contribute to increased pulmonary vascular resistance in accordance with observations in humans. We next measured thromboxane B_2_ (TXB_2_), a stable metabolite of TXA_2_, a potent vasoconstrictor, smooth muscle cell mitogen and platelet aggregator, that might also contribute to increase pulmonary vascular resistance after MCT administration. Moreover, thromboxane levels have been shown to increase in humans [4], rats [16] and mice [17] affected by pulmonary hypertension. TXB_2_ levels measured in BAL fluid collected at study endpoint, showed a tendency to increase in a similar fashion in WT and COX-2 KD mice after MCT (WT/saline: 92±24, range 35–166, n = 6; COX-2 KD/saline: 80±17, pg/ml, range 30–150, n = 6; WT/MCT: 387±218, range 117–2107, n = 9; COX-2 KD/MCT: 455±224 pg/ml, range 120–1437, n = 6, NS). Although the wide ranges of TXB_2_ levels in BAL fluids suggest that the degree of platelet activation within the lungs in response to MCT is variable, nonetheless thromboxane may contribute to pulmonary vessel occlusion and increase pulmonary vascular tone in MCT-treated mice.

### Prostacyclin and PGE_2_ in MCT-treated lungs

6-keto-PGF_1α_, the stable hydrolysis metabolite of PGI_2_, was measured in BAL fluid as an index of PGI_2_ production in the lungs. Levels of this prostanoid after 10 wk MCT treatment were more than 2.5-fold lower in COX-2 KD than WT mice (1207±456, n = 8 vs 3266±786 pg/ml, n = 10; p<0.05). Interestingly, levels in control COX-2 KD mice were significantly higher than control WT (8825±3617, n = 6 vs 4124±864 pg/ml, n = 6; p<0.05). These findings suggest that COX-2 KD mice produce high basal levels of PGI_2_ in the lungs, presumably from COX-1, an abundant source of PGI_2_ [42], despite systemic PGI_2_ biosynthesis in these mice being reduced ≈50% compared to WT mice [20], as measured by the main urinary metabolite 2,3-dinor-6-ketoPGF_1α_. However the capacity of the lungs to produce PGI_2_ is drastically reduced after 10 wk MCT treatment (≈85% reduction in 6-keto-PGF_1α_, p = 0.032 vs COX-2 KD/saline), when COX-2 expression is knocked down.

In contrast, levels of PGE_2_ did not change significantly in BAL at study end-point after MCT treatment or between WT and COX-2 KD groups of mice (WT/sal: 1545±540, n = 4; WT/MCT: 1520±278, n = 8; COX-2 KD/sal: 2430±259, n = 4; COX-2 KD/MCT: 1832±424 pg/ml, n = 5). Similarly, systemic PGE_2_ production, measured as the urinary stable metabolite PGEM (9,15-dioxo-11α-hydroxy-2,3,4,5-tetranor-prostane-1,20-dioic acid), did not differ significantly between treatment groups (data not shown).

### Right ventricular hypertrophy after MCT

Since chronic PH can lead to right ventricular hypertrophy and failure in response to increased vascular resistance in the pulmonary circulation, we measured right ventricular wall thickness in vivo by echocardiography and by histopathological analysis of heart sections at study end point. Right ventricular hypertrophy was not evident after ten weeks MCT treatment in comparison to saline-treated mice (WT/saline: 0.33±0.02 mm, n = 6; COX-2 KD/saline: 0.32±0.02 mm, n = 5; WT/MCT: 0.37±0.02, n = 10; COX-2 KD/MCT: 0.40±0.03, n = 7; NS). These results were confirmed post-mortem on H&E stained heart sections in which there were no significant cardiac morphological differences between treatment groups (data not shown).

### Pulmonary vascular remodeling

**Figure 2:**
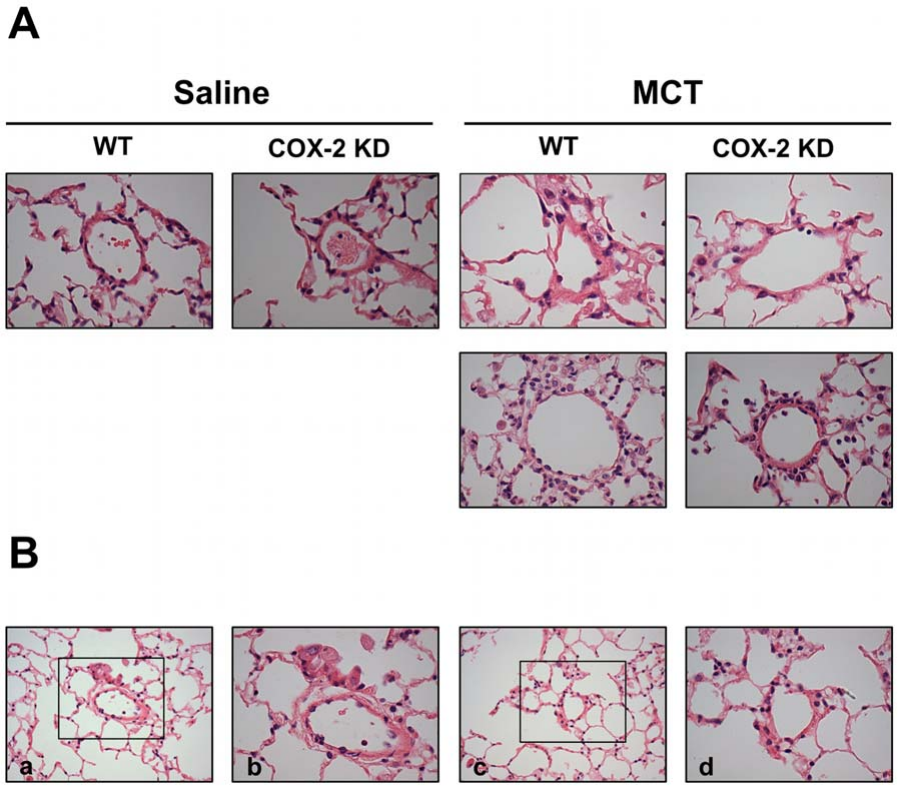
Minimal vascular pulmonary remodeling after MCT. A. Representative images 400x indicating only mild thickening of pulmonary arterioles from WT and COX-2 KD mice after MCT compared to saline. Lower panels are representative of WT and COX-2 KD MCT-treated mice that required euthanasia. Sections were stained with H&E. Lungs from 38 mice were evaluated microscopically with 2 lung sections/mouse, as follows: WT/saline, n = 6; COX-2 KD saline, n = 6; WT/MCT, n = 14; COX-2 KD/MCT, n = 12. B. Representative photomicrographs at 200x (a, c) of lung sections from a COX-2 KD MCT-treated mouse showing pulmonary mild perivascular edema with neutrophil infiltration in small arterioles. Boxed areas from a and c are shown at 400x in b and d.

Muscularization of resistive vessels, plexiform lesions and vasculitis leading to complete vessel obliteration have been described as hallmarks of pulmonary hypertension in humans and in animals, albeit in the latter with different degrees of severity depending on the model [32]. These morphological changes together with vasoconstriction, thrombotic events and inflammation, are known to contribute to the pathophysiology of PH. In our experimental conditions, both WT and COX-2 KD MCT-treated mice experienced a mild remodeling of pulmonary arterioles compared to saline-treated mice, with perivascular edema (**Figure 2**).

### Inflammation is minimal in MCT-treated WT and COX-2 KD mice

**Figure 3:**
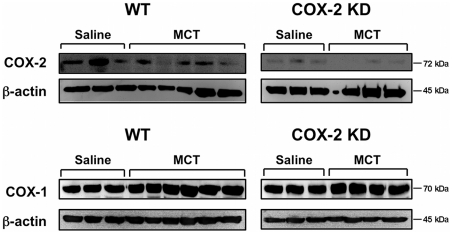
COX-1 and COX-2 expression in MCT-treated lungs. Western blot analysis for COX-2, COX-1 and β-actin expression in whole lung homogenates from WT and COX-2 KD mice treated with saline or MCT for 10 weeks. Each lane represents lung proteins from one mouse. WT/saline, n = 3; WT/MCT, n = 5-6; COX-2 KD/saline, n = 3; COX-2 KD/MCT, n = 3–4.

**Figure 4:**
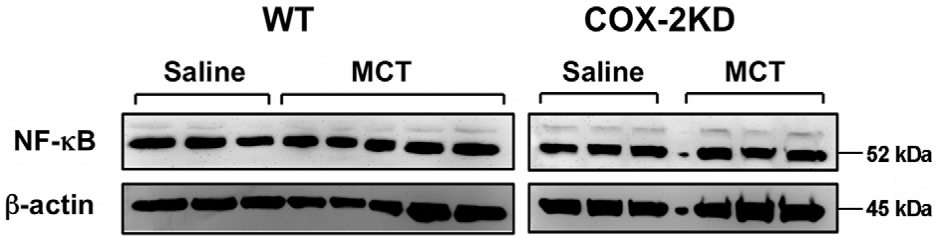
NF-κB expression in lungs is unchanged after MCT. Representative immunoblot for NF-κB p52 subunit with β-actin normalization in WT and COX-2 KD whole lung homogenates. WT/saline, n = 6; WT/MCT, n = 11; COX-2 KD/saline, n = 6; COX-2 KD/MCT, n = 7.

Since studies on animal models of PH and humans suggest that inflammation may play an important role in the pathogenesis of PH, we investigated the expression of several inflammatory genes and cytokines in whole lung homogenates, BAL fluid and plasma collected at study endpoint. We found that COX-2 was not significantly induced in MCT-treated mice compared to saline controls, as revealed by lung section immunolabeling (data not shown). Western blot analysis on whole lung homogenates showed that COX-2 protein expression was comparable between saline- and MCT-treated WT mice and drastically reduced in COX-2 KD mice (**Figure 3**). Similar results were obtained for COX-2 mRNA levels measured by quantitative PCR with no significant differences between saline- and MCT-treated groups (WT/saline: 0.79±0.1, n = 6; COX-2 KD/saline: 0.01±0.005, n = 6; WT/MCT: 1.02±0.14, n = 10; COX-2 KD/MCT: 0.03±0.02, n = 7; p<0.001 COX-2 KD vs WT). As expected, levels of COX-2 mRNA were reduced by > 90% in COX-2 KD lungs compared to WT. COX-1 protein expression was similar in WT and COX-2 KD lungs and remained constant after MCT (**Figure 3**). COX-1 and COX-2 levels in COX-2 KD mice are in accordance with the levels of expression previously found in other cell types and tissues [20]. Tumor necrosis factor-α (TNFα), a potent cytokine produced mainly by activated macrophages, was unchanged after MCT treatment in both WT and COX-2 KD mice (assessed in lung mRNA extracts and in BAL fluid, data not shown). Similar to TNFα, several other inflammatory cytokines (IL-6, IL-10, MCP-1, IFN-γ, IL-12p70) assessed by bead array on BAL and plasma samples, were below the detection limit of the assay (5–53 pg/ml) in all animals studied (WT/Saline n = 3, WT/MCT, n = 6; COX-2 KD/Saline n = 3, COX-2 KD/MCT, n = 3; data not shown). We next analyzed NF-κB protein expression since NF-κB activation is known to induce the transcription of inflammatory cytokines and proteins, including COX-2. As depicted in **Figure 4**, NF-κB subunit p52 was not significantly different in MCT-treated mice with no difference between WT and COX-2 KD mice. A qualitative differential cell analysis on bronchoalveolar lavages at study end-point revealed relative increases in percentage of neutrophils (WT/saline: 1.6±0.8%, n = 5 vs WT/MCT: 26.8±7.9%, n = 9; p<0.05; COX-2 KD/saline: 1±0.6%, n = 6 vs COX-2 KD/MCT: 18.7±4.6%, n = 6; p<0.05) and lymphocytes (WT/saline: 5.2±1.8%, n = 5 vs WT/MCT: 11.6±3.4%, n = 9; NS; COX-2 KD/saline: 5±1.7%, n = 6 vs COX-2 KD/MCT: 10.2±1.8%, n = 6; p<0.05) after MCT treatment. The relative number of monocytes was not significantly different between saline- and MCT-treated animals (data not shown). This is consistent with mild, ongoing pulmonary inflammation. This was also noted histologically, and characterized by mild perivascular edema and small increases, predominantly in neutrophils, within the alveolar interstitium and surrounding pulmonary arterioles (**Figure 2**). No differences were detected between WT and COX-2 KD animals treated with MCT and no histological evidence of thrombosis was noted.

### Expression of endothelial markers and oxidative stress (eNOS, PGIS, HO-1 and nitrotyrosine) in MCT-treated lungs

**Figure 5:**
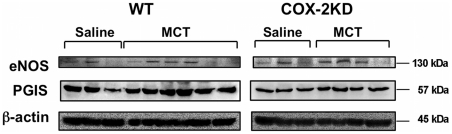
eNOS and PGIS expression in lungs after MCT treatment. eNOS and PGIS protein levels, measured in whole lung homogenates, were not significantly affected by MCT in WT and COX-2 KD mice in comparison to saline-treated mice. β-actin is shown for normalization. WT/saline, n = 6; WT/MCT, n = 11; COX-2 KD/saline, n = 6; COX-2 KD/MCT, n = 7.

MCT has been shown to induce megalocytosis, enlargement of the Golgi apparatus and block in mitosis of pulmonary endothelial [43] and epithelial cells [44] and, although the exact mechanism(s) by which MCT induces pulmonary damaging effects are not fully elucidated, endothelial injury within pulmonary vasculature is believed to be one of the most prominent. We therefore investigated the expression of several genes related to endothelial function. We found that lung expression of endothelial nitric oxide synthase (eNOS) and prostacyclin synthase (PGIS), the major source of two vasodilators NO and PGI_2_, produced by endothelial cells, was not significantly affected by MCT in both WT and COX-2 KD mice after 10-weeks of treatment (**Figure 5**).

**Figure 6:**
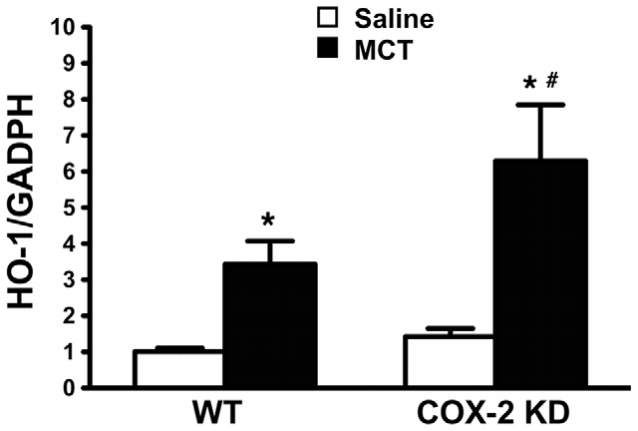
HO-1 mRNA is induced in response to MCT. HO-1 mRNA expression detected by quantitative RT-PCR in whole lung extracts. GADPH was used as endogenous control. Results were calculated as fold-change relative to reference cDNA, as described in Methods and expressed as fold-change relative to WT/saline. WT/saline, n = 6; WT/MCT, n = 10; COX-2 KD/saline, n = 6; COX-2 KD/MCT, n = 6. * p<0.05 vs saline; #, p<0.05 vs MCT-treated WT.

**Figure 7:**
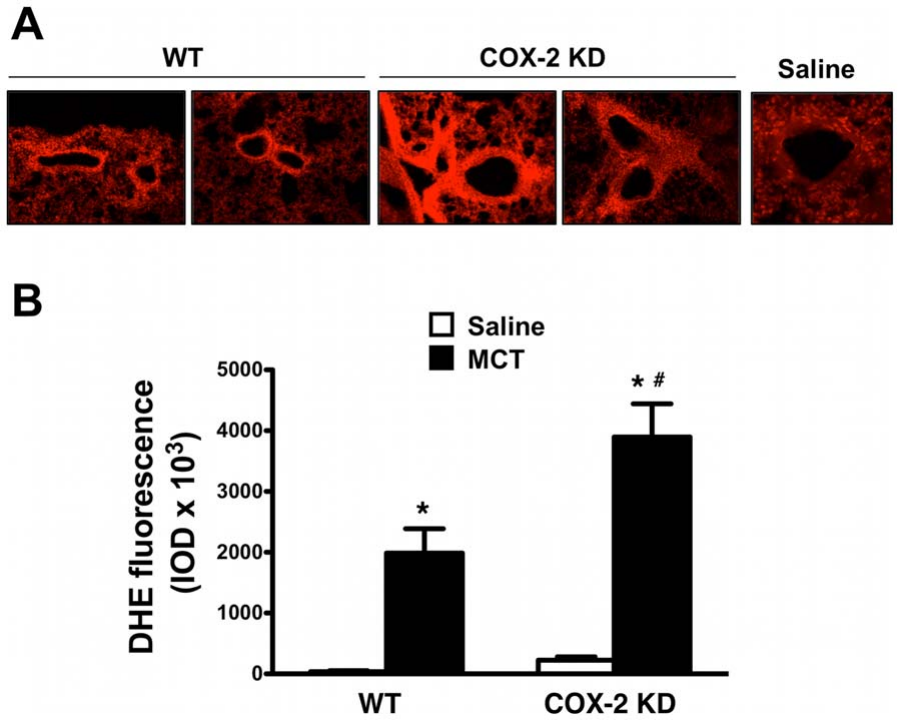
Oxidative stress is exacerbated in lungs from COX-2 KD mice after MCT treatment. A. Images from MCT-treated mice (20x objective) revealing intense DHE fluorescence in pulmonary arterioles. The image of a DHE-fluorescing arteriole (40x) from a saline control was included for comparison to indicate background fluorescence. B. Intensities of DHE fluorescence are summarized in graph format and expressed as integrated optical densities (IOD). Lung sections (n = 7–27) from 4–7 mice from each treatment group are represented as averaged fluorescence values. DHE, dihydroethidine. *, p<0.05 vs saline; #, p<0.05 vs MCT-treated WT.

**Figure 8:**
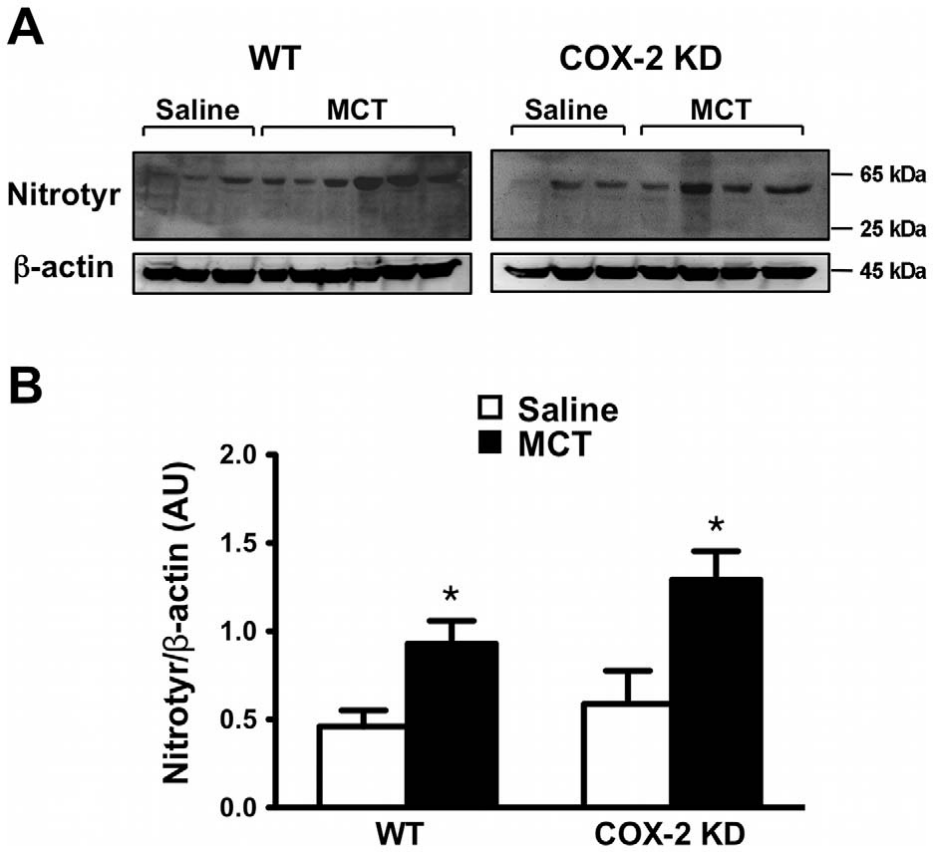
Oxidative stress is exacerbated in lungs after MCT treatment. A. Detection of nitrated tyrosine residues in whole lung homogenates in WT and COX-2 KD mice after MCT administration compared to saline-treated mice. The antibody detected a specific band (Nitrotyr) at ≅65 kDa. β-actin is shown as loading control. Each lane represents lung proteins from one mouse. B. Quantitation of band intensities expressed as ratio with β-actin, in arbitrary units (AU). WT/saline, n = 3; WT/MCT, n = 6; COX-2 KD/saline, n = 3; COX-2 KD/MCT, n = 4. * p<0.05 vs saline.

Heme oxygenase-1 (HO-1) is a heat shock protein induced in endothelial cells by a variety of stresses, including oxidative stress. HO-1 over-expression has been shown to play a defensive role in MCT-induced PH in mice [35]. In our experimental conditions, HO-1 mRNA from lung extracts, was induced by 3.4-fold in MCT-treated WT and by 5.7-fold in COX-2 KD mice compared to saline controls (**Figure 6**). HO-1 up-regulation at study endpoint suggests that MCT-treated lungs experienced a sustained oxidative stress during the treatment period. We therefore measured indirectly oxidative stress by DHE fluorescence of lung sections. MCT treatment dramatically increased this parameter in WT and COX-2 KD lung sections compared to saline treatment. DHE staining was particularly intense around pulmonary arterioles (**Figure 7A** and quantitation in **7B**). As another measure of oxidative stress in MCT-treated lungs, we assessed nitrotyrosine content in proteins from whole lung homogenates by Western blotting. As depicted in **Figure 8A** (quantitation in graph, **8B**), MCT treatment induced nitration of tyrosine residues in both WT and COX-2 KD mice.

**Figure 9:**
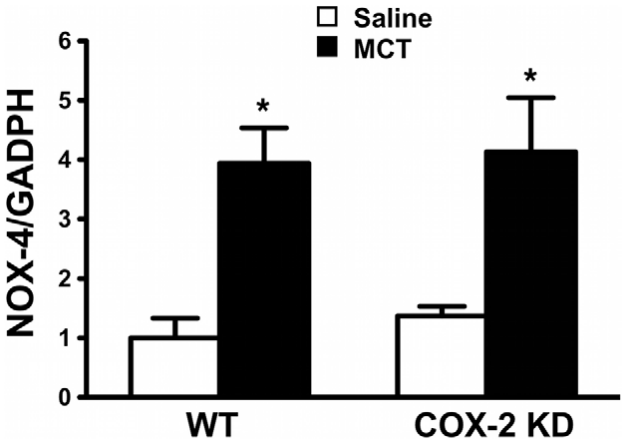
NOX-4 is induced in response to MCT. NOX-4 mRNA expression detected by quantitative RT-PCR in whole lung extracts. Results were expressed as fold-change relative to WT/saline after normalization to endogenous control GADPH. WT/saline, n = 5; WT/MCT, n = 9; COX-2 KD/saline, n = 4; COX-2 KD/MCT, n = 6) *, p<0.05 vs saline.

NADPH oxidase is considered a major source of superoxide anion in vascular tissues [45]. We found that NOX-4, a NADPH oxidase abundantly expressed in vascular smooth muscle cells [46] and endothelial cells [47], was upregulated by ≈ 4-fold in response to MCT (3.9±0.6 in WT/MCT, n = 9 and 4.1±0.9 in COX-2 KD/MCT, n = 7 compared to saline; **Figure 9**). Whole lung expression of NOX-2/gp91phox subunit, considered the predominant catalytic subunit of NADPH oxidase in phagocytic cells, and extracellular superoxide dismutase (EC-SOD), a known O_2_^.−^ scavenger, did not change significantly after MCT (data not shown). Taken together, these results suggest that sustained oxidative stress and endothelial dysfunction may contribute to the pathogenesis of MCT-induced PH and that in COX-2 KD lungs oxidative stress is exacerbated compared to WT.

## Discussion

In experimental animal models with COX-2 null mice and COX-2 inhibitors a protective role of COX-2 in the development of PH is indicated but the exact signaling pathways are poorly understood. Here we studied the effects of COX-2 modulation in MCT-induced PH using unique induced mutant mice, genetically manipulated to express ≈20% COX-2 (COX-2 KD). The advantage of COX-2 KD mice is that they mimic the administration of COX-2 selective inhibitors, i.e. incomplete suppression of COX-2 products, without severe phenotypic abnormalities, such as renal defects, associated with COX-2 null mice [48], [49]. In the attempt to identify the molecular mechanisms contributing to MCT-induced PH, we analyzed lung and heart samples from WT and COX-2 KD at study end point (10 wk), when hemodynamic changes in right ventricular systolic pressure (RVSP) and pulmonary arterial pressures, were evident but rather modest.

PH is a complex disease and, to date, the lack of reliable animal models that recapitulate all the features of human PH has limited the discovery of new therapeutics to treat this severely disabling disease. One of the main features of human PH is loss and pruning of pulmonary peripheral vessels and muscularization of small and medium pulmonary arterioles. These morphological changes are considered the hallmarks of PH in humans and current animal models and they are known to contribute to increased lung vascular resistance leading to an elevation in pulmonary arterial pressure. In our experimental conditions, MCT administration did not result in a pronounced remodeling of pulmonary resistive vessels and right ventricles, as usually occurs in advanced PH. Despite the absence of detectable morphological changes in lungs and heart and only modest increases in RVSP, we found that oxidative stress, as evidenced by remarkable HO-1 induction, superoxide production and increased expression of nitrated tyrosine residues in lungs was the paramount pulmonary effect of MCT in mice. Extracellular superoxide dismutase, a known antioxidant protein, did not change significantly in MCT-treated mice suggesting that there is an increase in superoxide production (measured by DHE fluorescence) rather than a decrease in scavenging capacity of the lungs in these settings. Moreover, in response to MCT we found a ≈2-fold induction of ETR-A, the major receptor responsible for the vasoconstrictive activity of endothelin-1, and an increase of vasoconstrictor and prothrombotic molecule TXA_2_ in BAL fluid, compared to saline-treated mice and with no significant difference between WT and COX-2 KD mice. Taken together, these results suggest that oxidative stress and increased pulmonary vasoconstriction, may both contribute to pulmonary vascular functional impairment leading to the modest hemodynamic changes observed in MCT-treated mice. These results are in accordance with several studies showing that endothelial dysfunction in pulmonary vasculature, disrupted balance of vasoactive substances (endothelin-1 and NO, among others) and impaired endothelium-dependent pulmonary artery relaxation are observed prior to vascular remodeling or plexiform lesions in humans with PH [50] and animal models [51], [52].

Several enzymes including NADPH oxidase, uncoupled eNOS, xanthine oxidase, those involved in mitochondrial respiratory electron transport, lipoxygenases, COX, myeloperoxidases and cytochrome P450 can contribute to the production of reactive oxygen species (ROS) in physiological conditions. NADPH oxidase and uncoupled eNOS activity are recognized as the most abundant source of ROS in vascular tissues in cardiovascular diseases characterized by endothelial dysfunction [53]. NOX-4, a constitutively active gp91phox/NOX-2 subunit homolog and primary source of NADPH oxidase catalytic activity and ROS generation in VSMC [46] and endothelial cells [47], was induced by 4-fold in the lungs of MCT-treated mice and may be responsible for the increased production of superoxide in these settings. We did not find significant changes in endothelial NOS (eNOS), in whole lung homogenates after MCT and no difference between WT and COX-2 KD mice. Despite unchanged overall eNOS protein levels, we cannot exclude that uncoupled eNOS activity may in part contribute, in addition to NOX-4, to increased superoxide production in response to MCT. Indeed, recent studies linked oxidative stress-derived endothelial dysfunction due to NADPH oxidase- and/or uncoupled eNOS-derived superoxide, to the pathogenesis of PH in humans [54], [55] and animal models [56], [57], [58], [59], [60].

NOX-2/gp91phox subunit, the NADPH oxidase catalytic moiety in phagocytic cells, such as neutrophils, was not affected by MCT treatment. The number of monocytes and levels of TNFα and several other inflammatory cytokines produced mostly by activated macrophages (IL-6, IL-10, MCP-1, IFN-γ, IL-12p70) were unchanged before and after MCT despite mild increases in the proportion of neutrophils and lymphocytes and perivascular edema. Similarly, PGE_2_ in BAL fluid did not change significantly after MCT treatment. Furthermore NF-κB, a transcription factor activated by inflammatory stimuli, remained unchanged after MCT in both WT and COX-2 KD mice. However, since our Western blot analysis was limited to whole lung homogenate with an anti-p52 subunit specific antibody, we cannot exclude that NF-κB phosphorylation and translocation into the nucleus and/or expression/activation of other subunits, such as p65, equally important for NF-κB transcriptional activity, could occur in response to MCT. Whether there is a contribution of inflammation to PH pathogenesis at an earlier time point, possibly in the first few days or weeks after MCT administration, as suggested by others [21], has not been addressed in the present study.

COX-2 KD mice revealed ≈50% reduction in PGI_2_ lung production after MCT in comparison to WT mice. We did not detect significant differences in COX-2 and COX-1 levels between saline- and MCT-treated mice by Western blot, qPCR or immunostaining of lung sections and, as expected, COX-2 mRNA and protein expression were severely abrogated (≈90%) in COX-2 KD mice. Since PGIS, the enzyme responsible for specific conversion of COX-derived PGH_2_ into PGI_2_, and COX-1 protein expression were not affected by MCT treatment, taken together these results are consistent with lung COX-2 being a major source of PGI_2_ in settings of PH. It is important to note that peroxynitrite (ONOO^−^), generated by the reaction of O_2_^.−^ with nitric oxide at diffusion-limited rates in settings of oxidative stress, can oxidize critical sulphydryl and thioether groups and lead to tyrosine nitration in numerous proteins, including PGIS, reducing their catalytic activity. In MCT-treated mice nitrated tyrosine content was increased compared to saline-treated mice and since in COX-2 KD mice oxidative stress was exacerbated compared to WT, PGIS nitration is likely to occur. Impaired PGIS activity in these mice, together with severe down regulation of COX-2, could both be responsible for the marked PGI_2_ reduction, despite no overall PGIS protein expression change in MCT-treated COX-2 KD lungs. Lastly, reduction of PGI_2_ generation in settings of oxidative stress and reduced COX-2 activity, as in COX-2 KD mice after MCT administration, could divert unmetabolized arachidonic acid and/or PGH_2_ to other lipid metabolites (HETEs, isoprostanes) that may also contribute to impaired endothelial-dependent vasorelaxation and vasoconstriction in pulmonary vasculature causing increased pulmonary vascular resistance. To this end, Zou et al. [61], [62] demonstrated that hypoxia-reoxygenation or angiotensin II caused PGIS nitration in bovine coronary arteries and not only reduced PGI_2_ generation but also triggered PGH_2_-induced vasospasms and thrombosis via TXA_2_ receptor activation. Whether PGIS nitration and diversion of arachidonic acid and/or PGH_2_ to vasoconstrictor lipid metabolites, that could impair pulmonary arterial relaxation, occur in response to MCT in COX-2 KD mice compared to WT will be the focus of future studies.

One major limitation of this study is that MCT, despite sustained pulmonary oxidative effects exacerbated by the lack of COX-2, unexpectedly induced only modest hemodynamic changes in mice. In our preliminary studies, MCT in the range 50–300 mg/kg BW for 4 wk was not effective in increasing right ventricular pressure and pulmonary arterial muscularization, despite a modest increase, without reaching statistical significance, in the 300 mg/kg BW-treated group. These pilot studies motivated us to increase the regimen of weekly MCT administration to 600 mg/kg BW for 10 wk in order to observe sustained pulmonary effects in mice. This dose is approximately 10-fold higher than the one commonly used in rats (60–75 mg/kg BW). Species-specific differences in hepatic enzymes essential for MCT transformation into the pyrrole active metabolite account for a well-known resistant phenotype of mice to MCT pulmonary effects compared to rats [26], [27]. Notably, MCT administered weekly at 600 mg/kg was in large part tolerated by WT mice (3 of 14 died) but caused duress in COX-2 KD mice (14 of 19 died or required euthanasia). Acute hepatic necrosis was evident in some of the MCT-treated mice that died or required euthanasia and it was more pronounced in COX-2 KD mice. Hepatic toxicity associated with MCT administration in experimental animals has been correlated with a reduction in glutathione and anti-oxidant levels in the liver [63]. The exact mechanisms by which low COX-2 levels increased MCT-induced hepatic toxicity are unknown but they may be related to microsomal PGE synthase (mPGES-1), an inducible glutathione-dependent enzyme of the MAPEG family, whose expression and activity are closely linked to COX-2 [64], [65]. Further studies will be necessary to investigate MCT-induced hepatic toxicity in COX-2 KD mice.

MCT at 600 mg/kg BW has recently been employed by several other investigators to induce PH in mice [28], [29], [30], [31], [35]; however, in our study, this MCT regimen caused only a mild increase in pulmonary arterial pressure in mice, a modest increase in vasoconstrictors and mild chronic inflammation, without evident pulmonary vascular or cardiac remodeling. Whether longer treatments with MCT at this dose will be required to induce severe pulmonary and cardiac morphological and hemodynamic changes in mice is not clear. However, the fact that 3 of 14 MCT-treated WT mice died during this study and revealed hepatic necrosis suggests that hepatic toxicity may limit the use of MCT at 600 mg/kg for more than 10 wk.

In conclusion, the present study supports the hypothesis that oxidative stress-induced endothelial dysfunction, vasoconstriction and increased tendency for platelet activation in pulmonary vasculature and mild inflammation, exacerbated by the lack of COX-2, are the major determinants of PH at early stages of the disease when vascular and cardiac remodeling are not still apparent. We propose that NOX-4 inhibition or other therapeutic interventions that limit oxidative stress may prevent the progression of PH while COX-2 inhibitors may be hazardous in early stages of the disease. Furthermore, our study underscores the difficulty of using MCT in mice as a model of PH, due to the narrow therapeutic window between pulmonary effects and hepatic toxicity and points out that novel animal models are needed to study the pathogenesis of this complex disease.

## Materials and Methods

### Study design

All animal procedures were approved by Queen's University Animal Care Committee (protocol Funk-2009-027). WT and COX-2 knock down (COX-2 KD) inbred mice (C57BL/6 genetic background selected by The Jackson Laboratory speed congenics panel and further back-crossed to >99% C57BL/6) were housed in the same room on a 12h light/dark cycle and had access to standard chow and water ad libitum. COX-2 KD mice are characterized by severely suppressed (80-90%) COX-2 expression, as previously described in detail [20]. Monocrotaline (MCT, Sigma-Aldrich) solution was freshly prepared by dissolution in warm saline and prepared to pH ≈7.0. WT and COX-2 KD mice (males/females, 8–10 weeks old) received either 10 µl MCT solution/g body weight (BW; 600 mg/kg), intra-peritoneally, once weekly for 10 weeks or saline. Body weights were recorded before each MCT or saline administration. Clinical condition, including any sign of distress was carefully monitored and recorded during the study.

### Echocardiography to assess right ventricular hypertrophy, pulmonary artery and left ventricular function

After the final MCT administration, all surviving mice underwent echocardiography analysis (VisualSonics Vevo770, Toronto, Canada). During the procedure, isofluorane/O_2_ administration was administered by facemask to keep mice lightly anesthetized with heart rates in the range of 400–500 bpm. The right ventricle was visualized in a right parasternal long axis view with a 704 RMV scan-head. Right ventricular wall thickness was measured from images acquired in M-mode, using the depth interval (mm) generic measurement tool (Vevo770 v3.0 software, VisualSonics). Doppler flow images were recorded from the left parasternal long axis view with the 707 B scanhead slightly pointing to the left shoulder to visualize the pulmonary artery. Volume measurement was acquired at the level of the pulmonary valve and several indices of pulmonary artery blood flow (velocity-time integral, mean and peak pressure gradient and mean and peak velocity) were assessed using the pulmonary valve protocol measurement tool. Left ventricular function and dimensions (cardiac output, stroke volume, ejection fraction, fractional shortening, left ventricular diameter in systole and diastole, left ventricular volume in systole and diastole) were measured with the LV wall trace measurement tool from M-mode images acquired from a left parasternal short axis view at the level of the papillary muscles.

### Measurement of right ventricular pressure

Right ventricular pressures were measured as an index of pulmonary artery pressure. Briefly, mice were anesthetized with sodium pentobarbital (32 µg/g BW), placed on a heating pad and mechanically ventilated through a 22-gauge cannula (120 breaths/min, Harvard Apparatus rodent ventilator). By pulling the hyphoid cartilage upwards, the thoracic cage was gently opened from the diaphragm and through the sternum to expose the heart. Tissue was cauterized when necessary to minimize any blood loss. The exposed heart was superfused with warm saline during the procedure. The tip of a 25G needle, previously immersed in heparin solution (Hepalean, 10,000 USP units/ml, Organon, Toronto, Canada), was inserted into the right ventricle by gently piercing the wall, using the right coronary artery as guide. The tip of a radio-telemetry pressure transducer (TA11PA-C10, Data Systems International, DSI) was inserted through the small aperture after needle retraction. Pressure waveforms were monitored in real-time using the “trace and save” setting in the continuous sampling acquisition mode (Dataquest ART system, DSI) and recorded for at least 10 min for each mouse. Right ventricular pressures were calculated by averaging 15 s intervals of continuous recording.

### Tissue harvesting

At the end of right ventricular pressure measurements, bronchoalveolar lavage (BAL) fluid was collected by two intra-tracheal washes, with 800 µl ice-cold PBS. BAL fluid was centrifuged at 1200 rpm for 8 min at 4°C to remove any cellular component and the supernatant stored at −80°C for prostanoid and cytokine analysis. The BAL pellet was resuspended in 0.5 ml PBS and 50 µl of cell suspension was cytospun (800 rpm, 4 min) onto Superfrost glass slides (Fisher Scientific) and used for differential cell count after Wright's staining. Heparinized blood was collected via cardiac puncture and plasma was separated by centrifugation at 2500 rpm for 10 min at 4°C. Heart and lungs were removed en-bloc and washed with PBS on ice. The right lung was removed and immediately immersed in RNAlater (Ambion), held at 4°C overnight and then stored at −80°C pending further analysis. The remaining left lung and heart were gravity-fixed overnight with 10% buffered formalin via an intra-tracheal 22G cannula. Hearts were sectioned transversely and immersed in 10% buffered formalin until preparation of sections and immunostaining to assess cardiac hypertrophy. Lungs were paraffin-embedded and processed as described below.

### Lung histopathology analyses

For routine microscopic evaluation lung lobes were embedded in paraffin blocks, sectioned and stained with hematoxylin-eosin (H&E). To assess pulmonary vascular remodeling and COX-2 expression after MCT treatments, 8 µm lung sections were prepared from paraffin-embedded lungs. To assure a standardized and unbiased comparison between animals, taking into account the complex branching structure of the lungs, longitudinal sections were prepared by systematic sampling at the 10^th^, 15^th^, 20^th^ and 25^th^ consecutive 8 µm interval for each animal (80, 120, 160 and 240 µm depth), using the pulmonary artery as hallmark. Lung sections were rehydrated in PBS and immunolabeled with a specific marker for smooth muscle (Actin, α-smooth muscle, Immunohistology kit, Sigma-Aldrich) or with a rabbit polyclonal anti-COX-2 antibody (Cayman Chemical #160126). COX-2 protein was detected with a Vectastain ABC kit (rabbit IgG) and DAB substrate following the manufacturer's instructions (VectorLabs). All sections were counterstained with H&E.

### Dihydroethidine fluorescence

Dihydroethidine (DHE) was used to assess superoxide anion (O_2_^. −^) levels in lung tissues as an index of oxidative stress, as described by others [57], [66], [67], [68], [69]. In the presence of O_2_^. −^, DHE is oxidized to ethidium, which intercalates with cellular DNA and gives a red fluorescent signal. Right lungs were frozen and kept at −80°C until OCT embedding and sectioning at −20°C (20 µm). DHE solution (10 mM) was freshly prepared in DMSO and diluted in PBS to 10 µM working solution. Lung sections were kept frozen until washed on ice with cold PBS and incubated with DHE solution at 37°C for 1 h. Sections were then washed in PBS and mounted with Permount. Digital images were captured with a Leica DM IRB microscope, a Q imaging digital camera and OpenLab 4.0.2 software. To assure consistency of staining, all lung sections were processed in the same day and imaging acquisition parameters (exposure time, gain and offset) were kept constant for all sections. For DHE quantitative analysis, fluorescence intensities were measured automatically by setting the threshold value to 180 on a color scale 0–256 (ImageProPlus 5.1) and expressed as integrated optical density (IOD). At least 3 images (696 x 520 pixels; 10x objective) from 2 different lung sections were acquired for each treatment group. Digital images were first quantified in a treatment-blinded fashion and then fluorescence values pertaining to the same treatment group were averaged. Intensity values below 180 represent background fluorescence from a saline-treated lung section used as reference.

### Western blot analysis

Whole lung homogenates were prepared by mechanical disruption on ice in a glass tissue grinder, with T-Per lysis buffer (Pierce) and freshly added protease inhibitor cocktail (Roche). Proteins were separated by 10% SDS-PAGE and transferred to PVDF membranes. After overnight blocking in 5% milk-TBS solution, membranes were incubated with the following primary antibodies: anti-COX-2 (Cayman Chemical, cat #160126), anti-COX-1 (Cayman Chemical, cat # 160109), anti-PGIS (Cayman Chemical, cat # 100023), anti-eNOS (AnaSpec, cat # 53458), anti-NF-κB (Santa Cruz, cat # sc-298), anti-nitrotyrosine (Cayman Chemical, cat # 10189540) and anti-β-actin (Sigma-Aldrich, cat # A5441). Protein bands were visualized by incubation with appropriate HRP-conjugated secondary antibodies and chemiluminescent reagent (GE-Amersham). Blot images were acquired with a FluorChem 8900 instrument (Alpha Innotech) and quantitated with ImageJ (www.nih.gov).

### Real-time PCR

Lungs, immersed in RNAlater (Ambion) immediately upon harvest to stabilize RNA, were homogenized in TRIzol (Invitrogen). Total RNA was extracted with chloroform and precipitated in isopropanol, as per the manufacturer's instructions. Total RNA (1 µg) was reverse-transcribed using iScript cDNA synthesis kit (BioRad). cDNA (150 ng) was added to 10 µl iTaq SYBR Green Supermix with ROX (BioRad) in the presence of 2 µl each of sense/antisense primers (200 µM final concentration). Optimal primer pairs were designed to span an intron-exon junction and to produce a short amplicon (≈150 bp) in order to maximize the specificity and efficiency of amplification reactions (PrimerExpress v.2 software). Primer sequences were as follows: endothelin-1 receptor-A forward 5′-CTTCCTGCAGAAGTCCTCCG-3′ and reverse 5′-TTCCTTGAACTCGGCTCCAG-3′; COX-2 forward 5′-AGCCAGGCAGCAAATCCTT-3′ and reverse 5′-ATTCCCCACGGTTTTGACA-3′; tumor necrosis factor-α (TNFα forward 5′- ATTCCTGCTTGTGGCAGGG-3′ and reverse 5′-GGTGGTTTGCTACGACGTGG-3′ and GAPDH forward 5′-CTGGAGAAACCTGCCAAGTA-3′ and reverse 5′-TGTTGCTGTAGCCGTATTCA-3′. The following primer sequences were kindly provided by Dr Lars Bellner (Department of Pharmacology, New York Medical College, Valhalla, NY, USA): HO-1 forward 5′-AAGCCGAGAATGCTGAGTTCA-3′ and reverse 5′- GCCGTGTAGATATGGTACAAGGA-3′; NADPH oxidase (NOX-2/gp91phox subunit: forward 5′-TGAATGCCAGAGTCGGGATTT-3′ and reverse 5′-CCCCCTTCAGGGTTCTTGATTT-3′ and NOX-4 subunit: forward 5′-GAAGGGGTTAAACACCTCTGC-3′ and reverse 5′-ATGCTCTGCTTAAACACAATCCT-3′); extracellular superoxide dismutase (EC-SOD) forward 5′-CCTTCTTGTTCTACGGCTTGC-3′ and reverse 5′-TCGCCTATCTTCTCAACCAGG-3′. Real time PCR reactions were carried out with an Applied Biosystems 7500 Real Time PCR instrument using the following thermocycler conditions: 95°C/1 min, 95°C/5 s and 60°C/45 s (40 cycles). Dissociation curve analysis (melting curve) following each amplification reaction and 1% agarose gel analysis confirmed the generation of primer-specific products. Relative quantification of RNA expression was determined using the Ct analysis settings of 7500 System Sequence Detection Software v.1.3 based on the ΔΔCt comparative method. In order to consistently compare samples analyzed in different days and assay plates, we used 1 µg cDNA prepared from a mouse total RNA reference sample, consisting of a pool of 11 mouse cell lines (Stratagene QPCR Mouse Reference Total RNA, cat. # 750600). Results were expressed as fold-change relative to this reference cDNA using GAPDH as endogenous control gene.

### Prostanoid and cytokine analysis

TXB_2_ and 6-keto-PGF_1α_ non-enzymatic metabolites of TXA_2_ and PGI_2_ respectively, and PGE_2_ were assessed in BAL by enzyme immunoassay (Cayman Chemical). Interleukin-6 (IL-6), interleukin-10 (IL-10), monocyte chemoattractant protein-1 (MCP-1), interferon-γ (IFN-γ), interleukin-12p70 (IL-12p70) and TNFα were analyzed in BAL and plasma samples using BD cytometric bead array, as per the manufacturer's recommendations (Mouse inflammation kit cat # 552364, BD).

### Cell count in bronchoalveolar lavage fluid

Wright's-stained cytospin preparations of air-dried bronchoalveolar lavage fluid were assessed microscopically, and the relative numbers of each cell type were determined and expressed as a percentage of the total cell population present.

### Statistical analysis

Data are expressed as mean±SE; Student's t-test was used for comparisons between different treatment groups; p values <0.05 were considered significant.

## Supporting Information

Figure S1 **Kaplan-Meier survival curves for MCT-treated WT (n = 14) and COX-2 KD (n = 19) mice.** Dashed line indicates no loss in survival of saline-treated WT (n = 6) and COX-2 KD (n = 6) mice. *, p = 0.0006.
(TIF)

Table S1 **Left Ventricular function is not altered by MCT treatment.** Indices of left ventricular function were measured in WT and COX-2 KD mice, treated with saline or MCT, from M-mode images of the left ventricle acquired by echocardiography, as described in Methods. CO, cardiac output; SV, stroke volume; EF, ejection fraction; FS, fractional shortening, LVDs, left ventricular diameter in systole; LVDd, left ventricular diameter in diastole; LVVs, left ventricular volume in systole; LVVd, left ventricular volume in diastole. Number of mice for each group is in parentheses. Mean±SE.
(DOCX)
