# Supplementary figures and images for: Pulmonary Oxidative Stress Is Increased in Cyclooxygenase-2 Knockdown Mice with Mild Pulmonary Hypertension Induced by Monocrotaline
Source: PLoS One. 2011 Aug 5;6(8):e23439. doi: 10.1371/journal.pone.0023439 (PMC3151294; doi:10.1371/journal.pone.0023439)

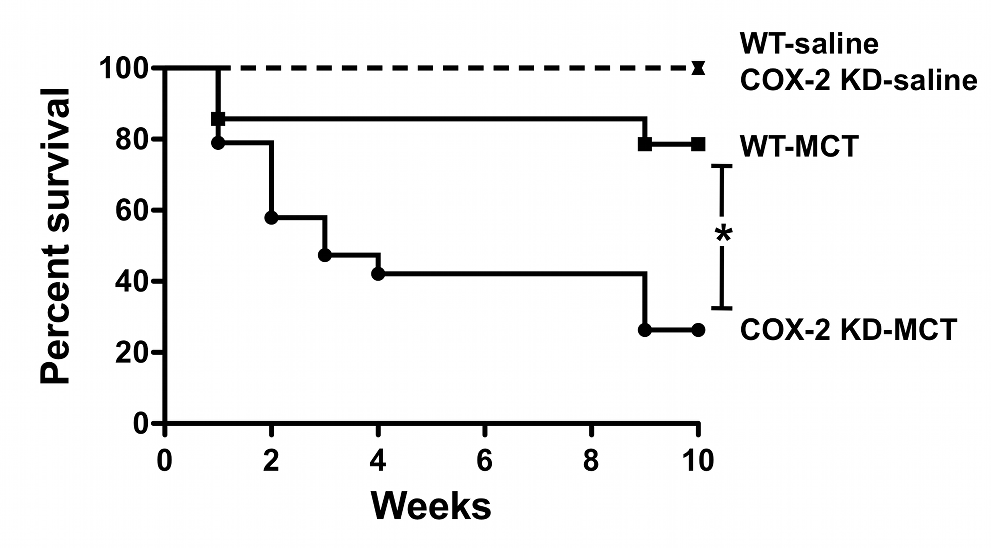

Supplement: Figure S1 — Kaplan-Meier survival curves for MCT-treated WT (n = 14) and COX-2 KD (n = 19) mice. Dashed line indicates no loss in survival of saline-treated WT (n = 6) and COX-2 KD (n = 6) mice. *, p = 0.0006. (TIF) [file pone.0023439.s001.tif]
