# Supplementary material for: Pulmonary Oxidative Stress Is Increased in Cyclooxygenase-2 Knockdown Mice with Mild Pulmonary Hypertension Induced by Monocrotaline
Source: PLoS One. 2011 Aug 5;6(8):e23439. doi: 10.1371/journal.pone.0023439 (PMC3151294; doi:10.1371/journal.pone.0023439)
Supplement: Table S1 — Left Ventricular function is not altered by MCT treatment. Indices of left ventricular function were measured in WT and COX-2 KD mice, treated with saline or MCT, from M-mode images of the left ventricle acquired by echocardiography, as described in Methods. CO, cardiac output; SV, stroke volume; EF, ejection fraction; FS, fractional shortening, LVDs, left ventricular diameter in systole; LVDd, left ventricular diameter in diastole; LVVs, left ventricular volume in systole; LVVd, left ventricular volume in diastole. Number of mice for each group is in parentheses. Mean±SE. (DOCX) [file pone.0023439.s002.docx]

**Table S1. Left Ventricular function is not altered by MCT treatment.**

|  | **WT/saline (n=4)** | **COX-2 KD/**  **saline (n=4)** | **WT/MCT (n=7)** | **COX-2KD/**  **MCT (n=6)** |
| --- | --- | --- | --- | --- |
| **CO (ml/min)** | **14.9 ± 0.9** | **11 ± 2.1** | **11.5 ± 1.3** | **12.4 ± 0.7** |
| **SV (μl)** | **35.9 ± 4.2** | **28.3 ± 5** | **35 ± 3.1** | **31.4 ± 1.6** |
| **EF (%)** | **82.3 ± 1.7** | **82.7 ± 4.1** | **81.8 ± 2.8** | **80.4 ± 3.7** |
| **FS (%)** | **50.4 ± 2** | **50.8 ± 4** | **49.8 ± 3** | **49.4 ± 4.6** |
| **LVDs (mm)** | **1.61 ± 0.02** | **1.49 ± 0.3** | **1.49 ± 0.1** | **1.61 ± 0.2** |
| **LVDd (mm)** | **3.26 ± 0.1** | **2.96 ± 0.3** | **3.0 ± 0.1** | **3.1 ± 0.1** |
| **LVVs (μl)** | **7.5 ± 0.25** | **7.2 ± 3.7** | **6.34 ± 1.1** | **8.2 ± 2** |
| **LVVd (μl)** | **43.3 ± 4.2** | **35.5 ± 9.2** | **34.9 ± 3.1** | **39.5 ±3.1** |

Indices of left ventricular function were measured in WT and COX-2 KD mice, treated with saline or MCT, from M-mode images of the left ventricle acquired by echocardiography, as described in Methods. CO, cardiac output; SV, stroke volume; EF, ejection fraction; FS, fractional shortening, LVDs, left ventricular diameter in systole; LVDd, left ventricular diameter in diastole; LVVs, left ventricular volume in systole; LVVd, left ventricular volume in diastole. Number of mice for each group is in parentheses. Mean±SE.
